# A framework for increasing scanner efficiency: the utility of a ‘Short aorta’ protocol to increase cardiac MRI capacity at the Yorkshire Heart Centre

**DOI:** 10.1093/ehjimp/qyag163

**Published:** 2026-10-06

**Authors:** Henry Procter, Jun Hui Tan, Lizette Cash, Marilena Giannoudi, Sindhoora Kotha, Sven Plein, Ananth Kidambi, Eylem Levelt, Anshuman Sengupta, Nicholas Jex

**Affiliations:** Department of Cardiology, Leeds Teaching Hospitals NHS Trust, Great George Street, Leeds LS1 3EX, United Kingdom; Department of Cardiology, Leeds Teaching Hospitals NHS Trust, Great George Street, Leeds LS1 3EX, United Kingdom; Department of Cardiology, Leeds Teaching Hospitals NHS Trust, Great George Street, Leeds LS1 3EX, United Kingdom; Department of Cardiology, Leeds Teaching Hospitals NHS Trust, Great George Street, Leeds LS1 3EX, United Kingdom; Biomedical Imaging Science Department, University of Leeds, Leeds Institute of Cardiovascular and Metabolic Medicine, Leeds LS2 9JT, United Kingdom; Department of Cardiology, Leeds Teaching Hospitals NHS Trust, Great George Street, Leeds LS1 3EX, United Kingdom; Department of Cardiology, Leeds Teaching Hospitals NHS Trust, Great George Street, Leeds LS1 3EX, United Kingdom; Biomedical Imaging Science Department, University of Leeds, Leeds Institute of Cardiovascular and Metabolic Medicine, Leeds LS2 9JT, United Kingdom; Department of Cardiology, Leeds Teaching Hospitals NHS Trust, Great George Street, Leeds LS1 3EX, United Kingdom; Department of Cardiometabolic Imaging, Baker Heart and Diabetes Institute, Melbourne, Australia; Baker Department of Cardiometabolic Health, University of Melbourne, Melbourne, Australia; Department of Cardiology, Leeds Teaching Hospitals NHS Trust, Great George Street, Leeds LS1 3EX, United Kingdom; Department of Cardiology, Leeds Teaching Hospitals NHS Trust, Great George Street, Leeds LS1 3EX, United Kingdom

**Keywords:** cardiovascular magnetic resonance imaging, aorta, scan optimization, service development

## Abstract

There are rising demands for cardiovascular magnetic resonance imaging (CMR) worldwide. Thoracic aorta assessment is a common CMR indication. There are many benefits to shorter scan times including improved patient experience, as well as efficiency of a service.

This service development project aimed to optimize the current ‘aorta’ protocol in a large tertiary CMR centre in the United Kingdom (which includes aortic valve [AV] and left ventricle [LV] assessment), with the aim to reduce scanning and reporting times. A ‘Short aorta’ protocol was devised (where only the thoracic aorta is assessed). The ‘Short aorta’ protocol requires a 30-minute scan slot, whereas the ‘Full aorta’ protocol (assessing thoracic aorta, AV and LV function) requires 60 minutes.

This project retrospectively analysed clinical and scan characteristics of patients referred to the Yorkshire Heart Centre for CMR before and after introduction of a ‘Short aorta’ protocol.

Over the two 13-week audit periods, thoracic aorta assessment was the main indication for 137 CMR referrals. Based on clinical indication, 95 (69%) could have used the ‘Short aorta’ protocol. After introduction of the ‘Short aorta’ protocol, 39 (46%) of 85 scans were protocolled as ‘Short aorta’. This resulted in significantly reduced scan times compared to the original ‘aorta’ protocol: 18 (IQR 15, 21) vs. 37 (IQR 32, 42) minutes; *P* = <0.0001).

Over the second audit period, 1.2 h of scanner time per week was saved. With appropriate protocolling and booking, 1.9 h of scanner time could be saved. This is significant capacity which can improve waiting times, offering better service for patients.

## Graphical Abstract

**Graphical Abstract:**
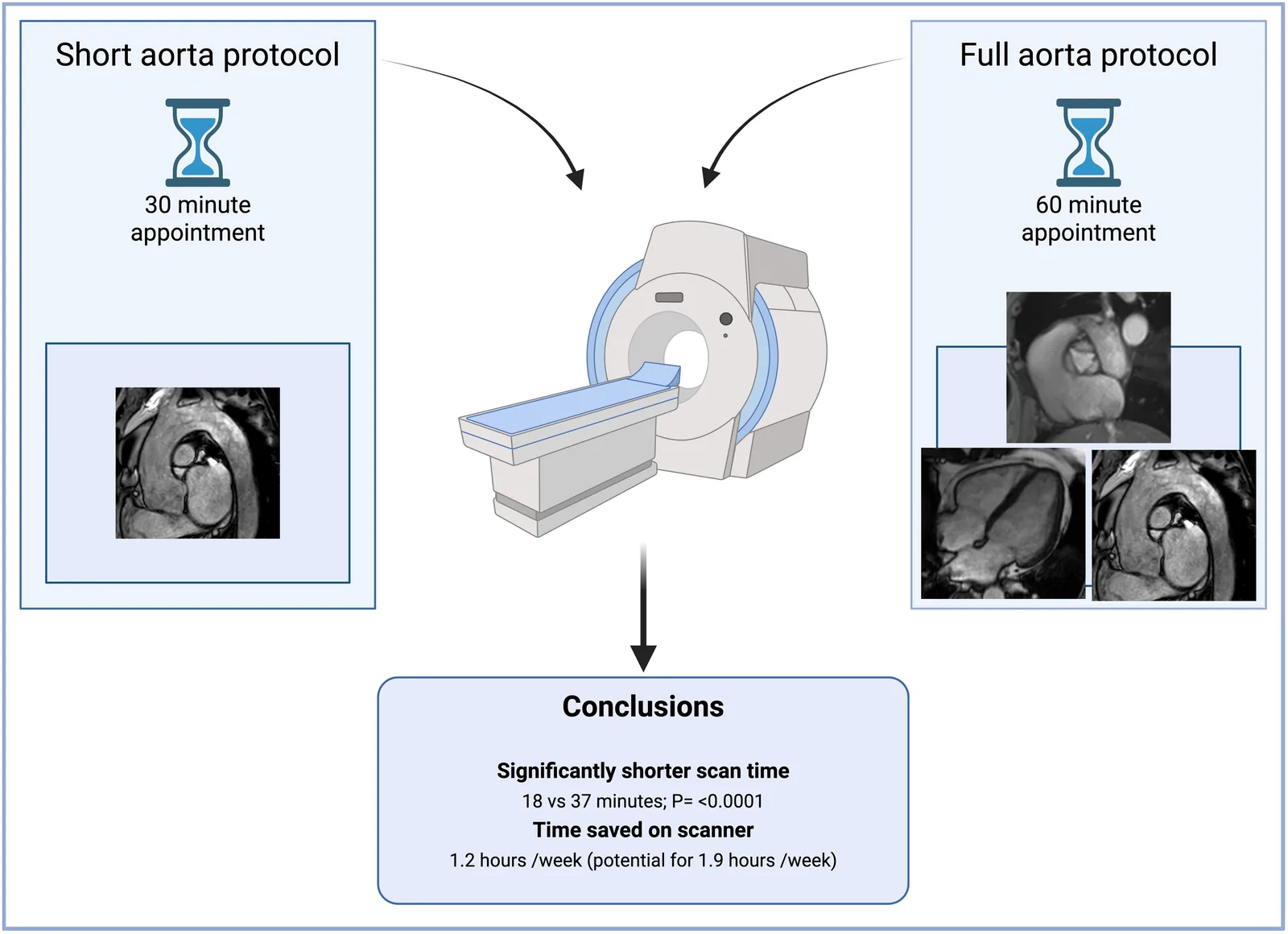

## Introduction

There is a rising demand for cardiovascular magnetic resonance imaging (CMR) worldwide, with growing evidence supporting its use across a wide range of diseases. There are many barriers to patients accessing CMR worldwide, including logistical shortfalls (which includes scanner shortages), financial implications of establishing CMR services, and a global shortage of scanning and reporting expertise.^1^ Patient factors known to limit CMR usage include claustrophobia, inability to lie flat or still for the scan duration, as well as anxiety caused by additional factors such as scanner noise, being left alone in the scanner room, and placement of the coils.^2^

There are over 100 000 CMR scans performed annually in the UK, with numbers rising each year.^3^ An important aspect of improving CMR provision is therefore improving daily patient throughput per scanner, while maintaining high quality scan images and reports.

One way to improve efficiency is to optimize how many sequences are acquired to answer the clinical question. Measurement of thoracic aortic dimensions is a common indication for CMR and allows measurement without exogenous contrast (avoiding the risk of contrast reactions). Conventionally, CMR protocols for imaging of the thoracic aorta include assessment of the aortic valve (AV) and the left ventricle (LV). However, AV and LV measurements are often not required to answer the clinical question.

This service development project aimed to compare the use of a ‘Full aorta’ protocol that includes LV and AV imaging, with a ‘Short aorta’ protocol that only assesses thoracic aortic measurements in a large teaching hospital in the United Kingdom.

## Methods

CMR scans at the Yorkshire Heart Centre are conventionally booked into 60-minute slots and the ‘Full aorta’ protocol includes LV and AV imaging. In March 2025, a new protocol was designed: ‘Short aorta’, which assesses the thoracic aorta without AV or LV assessment.

Both protocols include cine imaging of the aorta. Subsequent navigator-gated, free-breathing 3D whole-heart acquisitions in mid-diastole are performed. Field of view was 250 × 350 × 113 mm, using acquired voxel size of 1.25 × 1.25 × 2.4 mm, resulting in an acquisition matrix of 280 × 280 × 47. Images were reconstructed, giving a voxel size of 0.66 × 0.66 × 1.2 mm, and reconstructed matrix of 528 × 528 × 94. The following aortic measurements were reported: aortic annulus, sinus of Valsalva (SoV), sinotubular junction, ascending aorta, aortic arch, proximal descending aorta, and distal descending thoracic aorta. Area derived measurements were used, except SoV where sinus-to-sinus measurements were taken. The ‘Full aorta’ protocol then had additional cine imaging of the heart with AV flow imaging (*Figure 1*).

**Figure 1:**
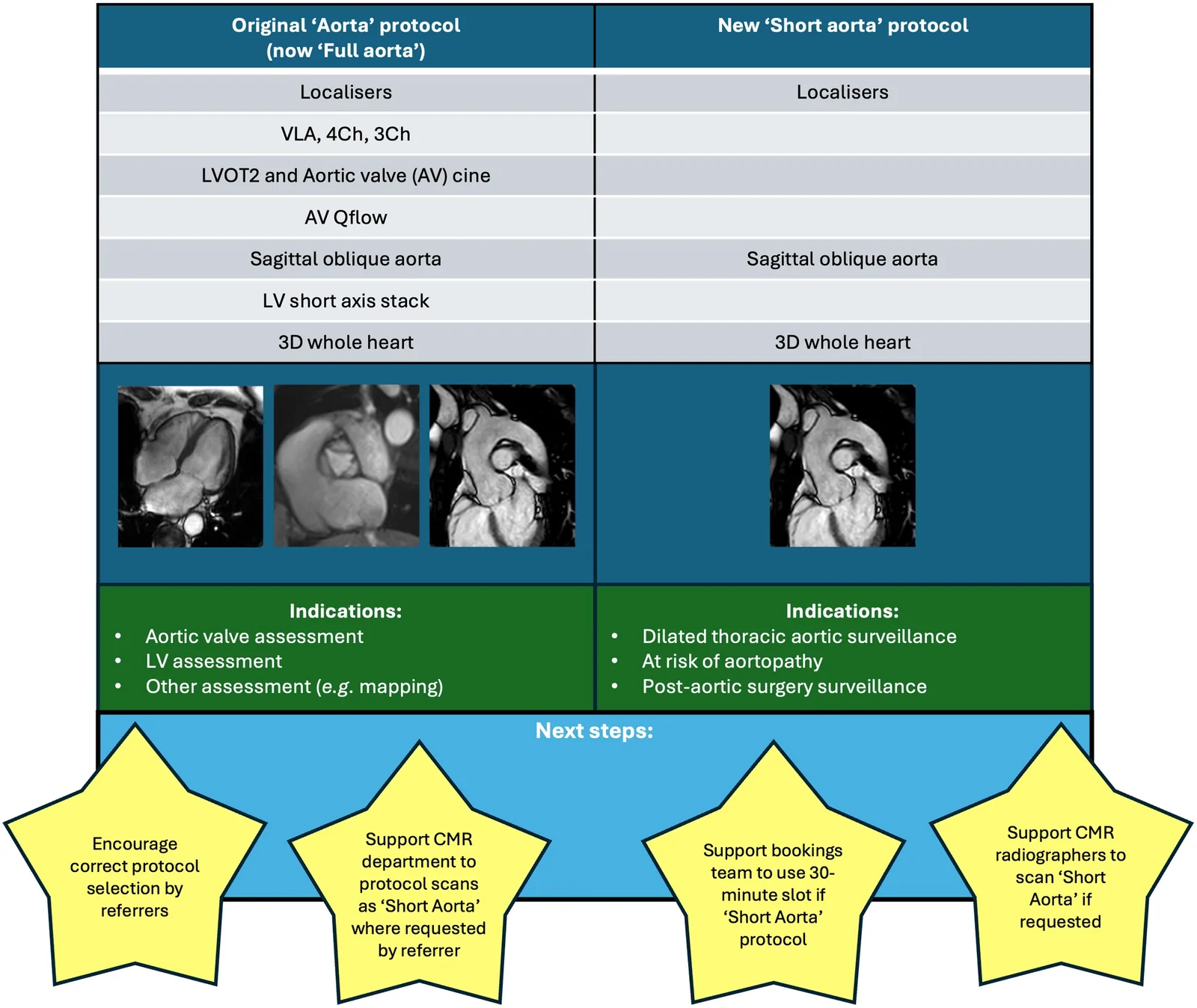
Summary of ‘Full aorta’ and ‘Short aorta’ protocols, with representative cine imaging.

A new requesting template allowed clinicians to choose a ‘Short aorta’ scan based on clinical indication. The ‘Short aorta’ protocol was indicated for the assessment of known thoracic aortic dilatation, patients at risk of aortopathy (including patients with bicuspid AV or family history of aortopathy), or post-operative assessment of patients following aortic surgery (*Figure 1*). Other common indications for CMR where assessment of thoracic aorta is required included assessment of AV disease, and assessment of LV size and function (typically in parallel with AV disease assessment). These indications for CMR would require a ‘Full aorta’ protocol.

A CMR clinician then protocolled CMR requests as either ‘Short aorta’, or ‘Full aorta’. The CMR booking team booked patient into appointments, with the intention that ‘Short aorta’ protocolled scans were given 30-minute slots.

Scans were performed on a Philips Ingenia 1.5T scanner, using a dStream Torso coil anteriorally, and integrated FlexCoverage posterior coil (each 32-channels).

The CMR radiographer scanned according to the pre-agreed protocols, unless there were unexpected findings needing protocol modification during image acquisition (including tissue characterization with T2-w imaging or late gadolinium enhancement. This was unlikely to occur as requirement for tissue characterization was often detected during protocol selection.

Demographics and imaging data were collected retrospectively through electronic records for patients referred to the Yorkshire Heart Centre (a regional tertiary cardiac centre) for CMR over two 13-week periods (08–10/2024 and 01–03/2026). Indications for CMR and time slot given for the scan on the CMR booking system were recorded. Scan results and duration were also recorded.

Statistical analysis was performed using GraphPad Prism (version 10.4.1). Data were checked for normality using Shapiro–Wilks testing, then presented as mean with 95% confidence intervals or median with interquartile range as appropriate. Categorical variables are expressed as counts (per cent). Comparisons were performed using the Student’s *t* test for normally distributed data and the Mann–Whitney U test for non-parametric data. A threshold of significance was defined as a two-sided *P-*value of <0.05.

## Results

The 2024 audit cycle, prior to introduction of the ‘Short aorta’ protocol, showed 52 CMR requests were protocolled to use the ‘Aorta’ protocol in this 13-week period. The most common indication was aortic dilatation surveillance, 32 (62%), with 3 (6%) assessing patients at risk of aortopathy, and 7 (13%) providing surveillance post aortic surgery (*Table 1*). All scans were given 60-minute slots, and scans took 37 (32, 42) minutes. Based on clinical indication, 81% of the aorta scans could have been ‘Short Aorta’ scans, had it been available.

**Table 1:** Summary of 2024 and 2025 audit findings

	2024 Audit: *n* = 52	2025 Audit: *n* = 85	*P* value
Age	54 (39, 64) Range: 19–72	59 (47, 65) Range: 17–83	
Female, *n* (%)	16 (31%)	25 (29%)	
Reason for referral			
Aortic dilatation (surveillance and formal assessment)^a^	32 (62%)	31 (36%)	
At risk of aortopathy	3 (6%)^a^	7 (8%)	
Post-aortic surgery (±AVR)^a^	7 (13%)	15 (18%)	
Aortic valve assessment	8 (15%)	17 (20%)	
LV assessment (with aortic assessment)	2 (4%)	15 (18%)	
Scans where could do ‘Short aorta’^a^	42 (81%)	53 (62%)	
Vetted as ‘Short aorta’	0 (0%)	39 (46%)	
2025 Audit only:			
Of scans protocolled as ‘Full aorta’ and patient attended for scan (*n* = 44), could they have been ‘Short aorta’?			
No—referrer asked for full, or needed full to answer the clinical question	—	34 (77%)	
Yes—referrer asked for short protocol (and ‘Short aorta’ was done on the day)	—	1 (2%)	
Yes—referrer asked for short protocol (and ‘Full aorta’ was done on the day)^a^	—	3 (7%)	
Yes—referral on old template (‘Short aorta’ not option for referrer)^a^	—	6 (14%)	
Time slot given:			
60 minutes	52 (100%)	53 (62%)	
30 minutes	0 (0%)	32 (38%)	
Scan length (minutes)			
‘Full aorta’	37 (32, 42)	38 (34, 46)	0.10
‘Short aorta’	—	18 (15, 21)	<0.0001*
Time saved:			
Over 13 week audit period	—	16 h	
Per week	—	1.2 h	
Potential scanner time saved if all appropriate scans scanned as ‘Short aorta’			
Over 13 week audit period	21 h	25 h	
Per week	1.6 h	1.9 h	

^a^Comparing ‘Short aorta’ protocol in 2025, to the ‘aorta’ protocol in 2024.

The 2025 audit cycle (after introduction of the ‘Short aorta’ protocol) showed 85 scans were requested with aortic assessment being the main indication for scanning in this 13-week period. Indications were similar, with 53 (62%) potentially suitable to be ‘Short aorta’ scans based on clinical indication (*Table 1*). Of the 85 aorta scans, 39 (46%) were protocolled as ‘Short aorta’ scans.

Of those scans protocolled as ‘Full aorta’ studies (*n* = 46), 44 patients attended and completed a CMR examination (two patients did not attend). Of these 44 scans, the ‘Full aorta’ protocol was either requested by the referrer, or was required to answer the clinical question in 34 (77%) of cases. In this real world service development project, there were an additional four (9%) scans where the referrer requested a ‘Short aorta’ protocol—but it was protocolled incorrectly by the CMR clinician. In one of these cases, the incorrect protocol was noted by the radiographer on the day of scanning, and the correct ‘Short aorta’ protocol was subsequently utilized. An additional 6 (14%) patients were referred using the old referral template (where the referrer was unable to select the ‘Short aorta’ protocol). Therefore potentially 49 (58%) of these scans could have used the shorter protocol.

Thirty-nine CMR requests were protocolled to use the ‘Short aorta’ protocol, with an additional scan incorrectly protocolled by the CMR clinician (but scanned using the correct protocol on the day by the radiographer). In total, 40 patients were scanned using the ‘Short Aorta’ protocol. There were no deviations to the protocol in any of these 40 scans, with the exception of one patient who was claustrophobic and so did not complete the study (this patient’s data were not analysed as part of the analysis of scan duration).

Where the ‘Short aorta’ protocol was used, the scan time was 18 (IQR 15, 21) minutes. This was significantly shorter than when the ‘Full aorta’ template was used (*P* = <0.0001).

Of those 39 scans protocolled as ‘Short aorta’ studies, 32(82%) were given a 30-minute slot. This discrepancy is due to administrative errors (booking patients into incorrect time slots, reflecting the real-world nature of this project). By utilizing shorter time slots, this saved 16 h of scanner time over the 13-week audit period (1.2 h per week). If all CMR requests were protocolled for ‘Short aorta’ where possible (based on clinical indication), and booked into 30-minute time slots, 25 h could potentially be saved over the 13-week audit period (1.9 h per week). This is consistent with findings from the 2024 audit cycle: if the ‘Short aorta’ protocol was used where possible in 2024 (based on clinical indication), this could have saved 21 h over the 13-week audit period (1.6 h per week).

## Conclusion

With ever increasing utilization of CMR in every day clinical practice, CMR departments need to constantly re-evaluate the service they can provide, to offer the best service for patients. This is important for patient comfort, but also for waiting times.

Based on scan indication, 53 (63%) of CMR studies in the 2025 audit could have potentially used the ‘Short aorta’ protocol. There are multiple steps in this process that need to be regularly reviewed to ensure this happens, starting with the requests being protocolled appropriately by the CMR clinician, booked into the appropriate time slot on the CMR scanner, and finally the abbreviated scan protocol used by the radiographer on the day of the scan.

The ‘Short aorta’ protocol has been shown to save 1.2 h per week on our CMR scanner (but with potential to save nearly 2 h per week). Next steps in this work are to work with requesting clinicians, CMR clinicians, radiographers (providing education on the benefit of shorter protocols for patients), and the bookings team (including the introduction of a CMR protocolling and booking standard operating procedures document). Further work might also include evaluation of CMR reporting times (however a shorter protocol would not necessarily translate to significant reductions, given advances in artificial intelligence and automatic contouring for basic ventricular and valvular flow assessments).

The ability to save nearly 2 h of scan time per week is significant capacity, which can be used to improve waiting times, to offer a better service for patients.

## Data Availability

The data underlying this article will be shared on reasonable request to the corresponding author.

## Lead author biography

Dr Henry Procter is a Cardiology Registrar and Clinical Fellow in Heart Muscle Disease at Leeds Teaching Hospitals NHS Trust. He graduated from the University of Leeds in 2014 and has been a cardiology trainee in the Yorkshire and Humber region since 2019. He has recently been awarded his PhD in 2025, funded through the National Institute for Health and Care Research Leeds BRC, under the supervision of Professor Eylem Levelt.

His doctoral research focused on the use of cardiac MRI and MR spectroscopy to assess myocardial health in patients with diabetes and severe aortic stenosis. His wider clinical and academic interests include the role of imaging in the assessment of patients with diabetes, heart failure, and inherited cardiac conditions. Alongside this, he has a strong interest in improving efficiencies within healthcare systems and optimizing current clinical pathways to enhance patient care and service delivery.
